# Delirium in Pediatric Intestinal, Liver, and Renal Transplantation*

**DOI:** 10.1097/PCC.0000000000003566

**Published:** 2024-09-05

**Authors:** Jan N.M. Schieveld, Jacqueline J.M.H. Strik

**Affiliations:** 1 Division of Child and Adolescent Psychiatry and Psychology, Department of Psychiatry and Psychology, Maastricht University Medical Center, Maastricht, The Netherlands.; 2 Department of Psychiatry and Neuropsychology, School for Mental Health and Neuroscience, European Graduate School of Neuroscience (EURON), Maastricht, The Netherlands.; 3 School for Mental Health and Neuroscience (MHeNS), Maastricht University, Maastricht, The Netherlands.; 4 Department of Pediatric and Youth Mental Health, Mutsaersstichting, Pediatric Mental Care Institution, Venlo, The Netherlands.; 5 Department of Psychiatry and Neuropsychology, Faculty of Health, Medicine and Life Sciences (FHML), Maastricht University, Maastricht, The Netherlands.

**Keywords:** phenotype, pediatric, pediatric delirium, screening

The first successful kidney transplant was performed by Dr. Joseph Murray in 1954 in adult identical twins. The first pediatric kidney transplant took place in 1960 (1) and now in the United States, each year, there are more than 1000 new pediatric kidney transplant candidates (2). The field of transplantation medicine and chronic immunosuppression has clearly come a long way. Even so, two questions about outcome remain. First, there are issues like the time course of posttransplant neuropsychological developmental, and subsequent academic skill attainment and quality-of-life. Second, there are factors related to new complications that arise, such as de novo lymphoproliferative disorders. In our view, one aspect of this landscape is the onset, time course, and complications of transplant-related pediatric delirium (PD).

PD is a neuropsychiatric condition that occurs secondary to a general medical or critical care condition and should be considered a serious complication of physical illness (3). At all ages, PD is associated with poor prognosis, as reflected by prolonged hospital stay, worse cognitive and functional outcomes, and higher mortality rate (3–9). Now, in this issue of *Pediatric Critical Care Medicine* (*PCCM*), Patel et al (10) report the prevalence of PD and possible risk factors in children who had intestinal, liver, or renal transplants. The authors hypothesized that PD was highly prevalent in such patients during the immediate postoperative period. They also aimed to compare risk factors for PD within and between the transplant groups.

The merits of the new study (10) are that it has some important strengths and the authors also highlight limitations for readers. For example, the strengths include: 1) the study’s power with sufficient numbers in each of the clinical groups, 2) an analysis of major risk factors for PD, especially young age, 3) risk stratification assessment using the pediatric index of mortality to distinguish between patient/groups of different severity, and 4) application of a range of mental state assessments such as an approximation to the Richmond Agitation-Sedation Scale, the State Behavorial Scale (SBS), and the Cornell Assessment of PD.

Regarding the limitations, in addition to the ones identified by Patel et al (10), we also consider it worth reflecting on three more as we think ahead to future work by our community. First, it is unclear how the distribution of age has been handled in the analysis. There is a broad range (0–17 yr), and the transplant groups differed in mean age (4, 2, and 9 yr). Also, the severity of illness scores differed, with the highest scores being prevalent in the youngest patient groups. So, one unanswerable question in the retrospective case series is whether the presence of PD is coupled with younger age, or with severity of illness, or both. The authors concluded that the severity of illness was not related to the presence of PD, but from other studies, we know that the severity of illness is a risk factor for PD (6–9). Large, multicenter, prospective work will be needed to resolve this issue.

Second, in the current “convenience sample” report (10), more than 60% of PD diminished within 3 days. At the time of practice to which the study refers—2016 to 2022—there was no screening for PD in the immediate postoperative period. Hence, potential misclassification or inclusion of emergence delirium cases in the PICU series cannot be unpicked retrospectively. This potential risk could explain why there seemed to be more agitated types of PD in the current report, whereas a 2020 review of the literature concludes that hypoactive forms of PD are more prevalent (11).

Third, when we review data from children who are unable to speak and express themselves, it is difficult to distinguish between the information that the SBS score is telling us and the state of “agitation and discomfort.” In our view, not every state of agitation in the PICU is PD—over 10 years ago we presented a flowchart to help practitioners (12)—and there exists an important and broad differential diagnosis mimicking PD (e.g., discomfort caused by pain, ventilation, hunger, thirst, itching, and having a full bladder, and so on). There are also potential consequences for treatment. Hyperactive delirium can be treated with antipsychotics, or, in case of severe or refractory agitation with rapid tranquilization; the efficacy of treatment of the acute hypoactive state on the other hand remains unproven (3).

So, to conclude, the report about posttransplant PD in this issue of *PCCM* moves the field forward and is important. Hopefully, future work can focus on PD-type specifying, and the interventions needed to improve outcomes for pediatric transplant patients and their families.
